# Proteomic-based stratification of intermediate-risk prostate cancer patients

**DOI:** 10.26508/lsa.202302146

**Published:** 2023-12-04

**Authors:** Qing Zhong, Rui Sun, Adel T Aref, Zainab Noor, Asim Anees, Yi Zhu, Natasha Lucas, Rebecca C Poulos, Mengge Lyu, Tiansheng Zhu, Guo-Bo Chen, Yingrui Wang, Xuan Ding, Dorothea Rutishauser, Niels J Rupp, Jan H Rueschoff, Cédric Poyet, Thomas Hermanns, Christian Fankhauser, María Rodríguez Martínez, Wenguang Shao, Marija Buljan, Janis Frederick Neumann, Andreas Beyer, Peter G Hains, Roger R Reddel, Phillip J Robinson, Ruedi Aebersold, Tiannan Guo, Peter J Wild

**Affiliations:** 1 ProCan, Children’s Medical Research Institute , Faculty of Medicine and Health, The University of Sydney, Westmead, Australia; 2 iMarker Lab, Westlake Laboratory of Life Sciences and Biomedicine, Key Laboratory of Structural Biology of Zhejiang Province, School of Life Sciences, Westlake University , Hangzhou, China; 3 Institute of Basic Medical Sciences, Westlake Institute for Advanced Study, Hangzhou, China; 4 Urology & Nephrology Center, Department of Urology, Clinical Research Institute, Zhejiang Provincial People’s Hospital, People’s Hospital of Hangzhou Medical College, Hangzhou, China; 5 Department of Pathology and Molecular Pathology, University Hospital Zürich, Zürich, Switzerland; 6 Department of Urology, University Hospital Zürich, Zürich, Switzerland; 7 Department of Urology, Cantonal Hospital Lucerne, Lucerne, Switzerland; 8 IBM Zürich Research Laboratory, Zürich, Switzerland; 9 State Key Laboratory of Microbial Metabolism, Joint International Research Laboratory of Metabolic and Developmental Sciences, School of Life Sciences and Biotechnology, Shanghai Jiao Tong University, Shanghai, China; 10 Empa - Swiss Federal Laboratories for Materials Science and Technology, St. Gallen, Switzerland; 11 Swiss Institute of Bioinformatics, Lausanne, Switzerland; 12 CECAD, University of Cologne, Cologne, Germany; 13 Department of Biology, Institute of Molecular Systems Biology, ETH Zürich, Zürich, Switzerland; 14 Faculty of Science, University of Zürich, Zürich, Switzerland; 15 Goethe University Frankfurt, Dr. Senckenberg Institute of Pathology, University Hospital Frankfurt , Frankfurt am Main, Germany; 16 Frankfurt Institute for Advanced Studies, Frankfurt am Main, Germany

## Abstract

Gleason grading is an important prognostic indicator for prostate adenocarcinoma and is crucial for patient treatment decisions. However, intermediate-risk patients diagnosed in the Gleason grade group (GG) 2 and GG3 can harbour either aggressive or non-aggressive disease, resulting in under- or overtreatment of a significant number of patients. Here, we performed proteomic, differential expression, machine learning, and survival analyses for 1,348 matched tumour and benign sample runs from 278 patients. Three proteins (F5, TMEM126B, and EARS2) were identified as candidate biomarkers in patients with biochemical recurrence. Multivariate Cox regression yielded 18 proteins, from which a risk score was constructed to dichotomize prostate cancer patients into low- and high-risk groups. This 18-protein signature is prognostic for the risk of biochemical recurrence and completely independent of the intermediate GG. Our results suggest that markers generated by computational proteomic profiling have the potential for clinical applications including integration into prostate cancer management.

## Abstract (precis)

This study demonstrates the ability of a protein-based signature to risk-stratify prostate cancer patients with Gleason grade groups 2 and 3 and predict the risk of biochemical recurrence.

## Introduction

Prostate cancer (PCa) is the third most common cancer among men by incidence (14.1%) and the fifth in terms of cancer-related mortality worldwide (among men, 7%) (1). In Australia, Western Europe, and North America, PCa is the most commonly diagnosed cancer among men and the second most common cause of cancer-related death (1). PCa is a highly heterogeneous disease, and so far, most of the treatment-decision algorithms depend on risk stratification based on the tumour stage, the prostate-specific antigen (PSA) level at the time of diagnosis, and the Gleason grade group (GG) (2). Although this clinical risk stratification has been shown to be of the prognostic and predictive value (3), better biomarkers are still required to improve patient stratification.

The Gleason score (GS) is a grading classification of the growth pattern of prostatic adenocarcinoma. The total GS (from 6 to 10) represents the summation of the two most common predominant scores (from 1 to 5) within the specimen (4). Despite its proven prognostic value, there was major heterogeneity within the GS7, with a differential prognosis observed between the GS7 (3 + 4) and GS7 (4 + 3) patterns (5). Because of this, the International Society of Urological Pathology (ISUP) developed a modification to the GS system in 2014 and created a new grading of five groups, with the aim of differentiating the GS7 (3 + 4) (termed GG2 in ISUP 2014) from the GS7 (4 + 3) (GG3) (6). The prognostic value of the GG system was validated in multiple cohorts, although its accuracy did not significantly differ from the older GS system (7). In addition, for the new GG system there is controversy regarding the value of incorporating the percentage of the GS4 within the GG2 and GG3, among other questions (8). This was addressed in the ISUP 2019 modification for PCa grading, which recommends reporting the percentage of GS4 patterns in any GG2 or GG3 case (9). Despite all of these modifications, both the GS and GG systems still have several limitations, including relatively long processing time, subjectivity, inter-observer variability, and unsatisfactory prediction of outcomes (10, 11, 12, 13, 14, 15, 16).

Therefore, there is a need to develop better prognostic biomarkers that can be interpreted either alone or when integrated with clinicopathologic features. There have been several ongoing efforts that aim to identify better molecular- and genetic-based prognostic biomarkers. These include metabolomic-based biomarkers (17), mRNA-based biomarkers such as SelectMDx and ExoDx Prostate IntelliScore, urine biomarkers such as PCA3, and genetic tissue–based biomarkers such as Oncotype DX, Confirm MDx, Prostatype (18, 19, 20), and Prolaris (21). Of note, only PCA3 and Prolaris are FDA-approved for specific indications (21). More recently, Proclarix showed better accuracy in detecting clinically significant PCa compared with free PSA percentage alone (22), with its utility in clinical practice yet to be confirmed.

During the last decade, proteogenomics has revealed a range of intra-patient network effects across multi-omic layers (15), and has described novel regulated pathways that are related to PCa progression (23) and PCa aggressive phenotypes (24, 25). Proteogenomics appears to have the potential to provide a deep and dynamic interpretation of the underlying pathways related to cancer development, classification, and progression (26). However, the lack of robust proteomic analyses of large cancer cohorts (27) has limited the incorporation of proteomic-based biomarkers into clinical practice (28, 29).

To address this limitation, we have compiled a cohort of 290 patients procured from the Prostate Cancer Outcomes Cohort Study (30) to generate large-scale proteomic measurements of PCa tissue samples using data-independent acquisition mass spectrometry (DIA-MS). The data have been analysed through purpose-built computational workflows at the Australian Cancer Research Foundation International Centre for the Proteome of Human Cancer (ProCan) in Westmead, Australia (31, 32, 33, 34, 35, 36, 37). We have identified differentially expressed proteins and pathways involved in PCa development and biochemical recurrence (BCR), including the identification of possible new therapeutic targets. Furthermore, we have built a protein-based signature, which showed better prognostic power than the GG and was completely independent of it.

## Results

### Proteomes of prostate tissue samples

**Figure 1.**
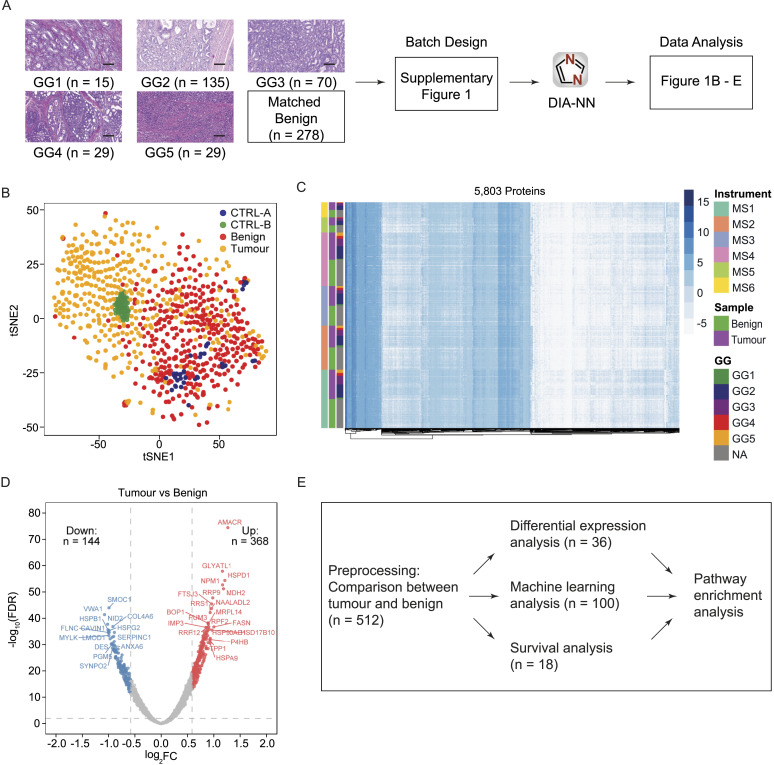
Proteomic analysis of PCa samples. **(A)** Overview of the study design. The dataset consists of prostatic tumour and matched benign tissue samples from 278 patients. Proteomic data were collected for 277 tumour samples and 278 benign samples in duplicate from 278 patients. A total of 1,475 MS runs were analysed in 31 batches, including tumour, benign, CTRL-A, and CTRL-B samples. The raw proteomic data were analysed by DIA-NN, quantifying 5,803 proteins. Scale bar = 100 μm. **(B)** tSNE projection of protein data superimposed with colour annotation of sample types. **(C)** Heatmap representation of the protein matrix with samples shown on the y-axis and proteins shown on the x-axis. The protein intensities were sorted first by the mass spectrometers, followed by tissue types and GGs. MS1–MS6 indicate the six mass spectrometers. **(D)** Volcano plot showing the up-regulated (n = 368) and down-regulated (n = 144) proteins in tumours with fold change (FC) > 1.5 and < 0.67 and the Benjamini–Hochberg (BH)-adjusted *P* < 0.01. Significant proteins are presented in red and blue colours, whereas other proteins are coloured in grey. **(E)** Analysis pipeline employed in this study and the number of proteins identified in each analysis. A total of 512 tumour-enriched proteins were identified from the comparison between tumour and benign samples, followed by stratification of the GG2 and GG3 using differential expression analysis and machine learning, and identification of a prognostic signature using survival analysis. Finally, pathway enrichment analyses were conducted for the significant sets of proteins.

**Figure S1.**
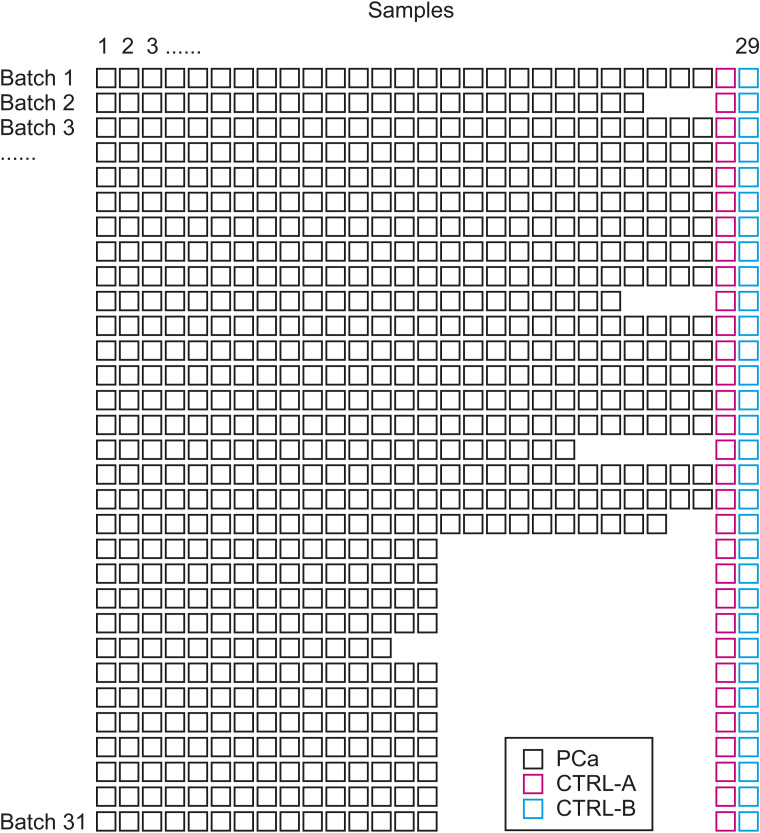
PPP1 study design. Each row indicates a batch, and each column indicates PCa tissue samples. Each batch contained between 15 and 29 PCa samples, one CTRL-A, and one CTRL-B.

**Figure S2.**
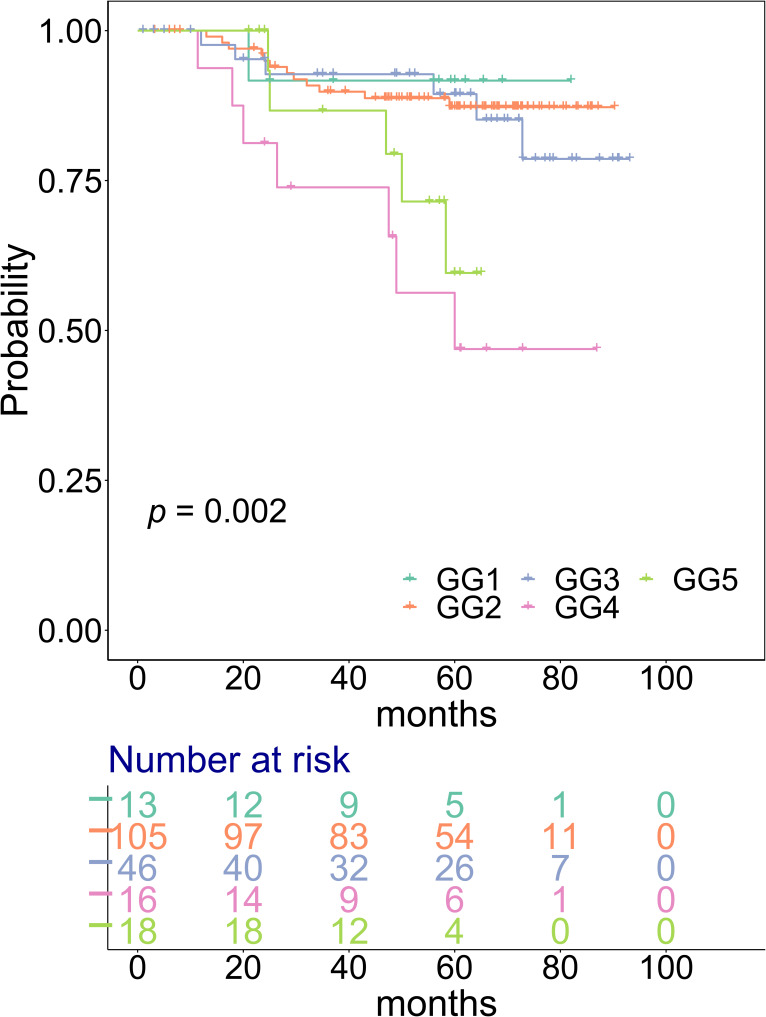
Kaplan–Meier (KM) curves for biochemical recurrence–free survival (BCRFS) for different GGs. Vertical lines illustrate patients who were censored at the time of their last clinical follow-up visit. The *P*-value shows the significance of the difference between survival estimates evaluated by the log-rank test. Coloured values represent the number of patients in each group under risk.

A total of 290 PCa patients representing the full range of GGs from the GG1 to GG5 were selected from the Prostate Cancer Outcomes Cohort Study retrospective cohort (30). Proteomes of 1,348 matched tumour and benign prostatic hyperplasia tissue sample runs from 278 patients were acquired and analysed with 12 patients being removed due to quality control (QC) steps. In each of the 31 batches, two controls (CTRL-A and CTRL-B) in duplicate were added to investigate technical variation, control quality, and assess reproducibility (Fig 1A; see the Materials and Methods section; Table S1; Fig S1). In this cohort, 198 of 278 patients had BCR data with a median follow-up of 59 mo and the range of time to recurrence is 11.37 to 72.8 mo (Table S1; Data Availability). Overall, most patients belong to the GG2 (n = 135), followed by the GG3 (n = 70; Fig 1A). Although there was a significant difference in outcome for GGs (*P* = 0.002), no significant difference was observed between the GG2 and GG3, and the GG4 unexpectedly showed the worst prognosis compared with all other GGs (Fig S2), reflecting the limitations of the GG system.

Table S1. Clinicopathologic features of patients with prostate cancer.

**Figure S3.**
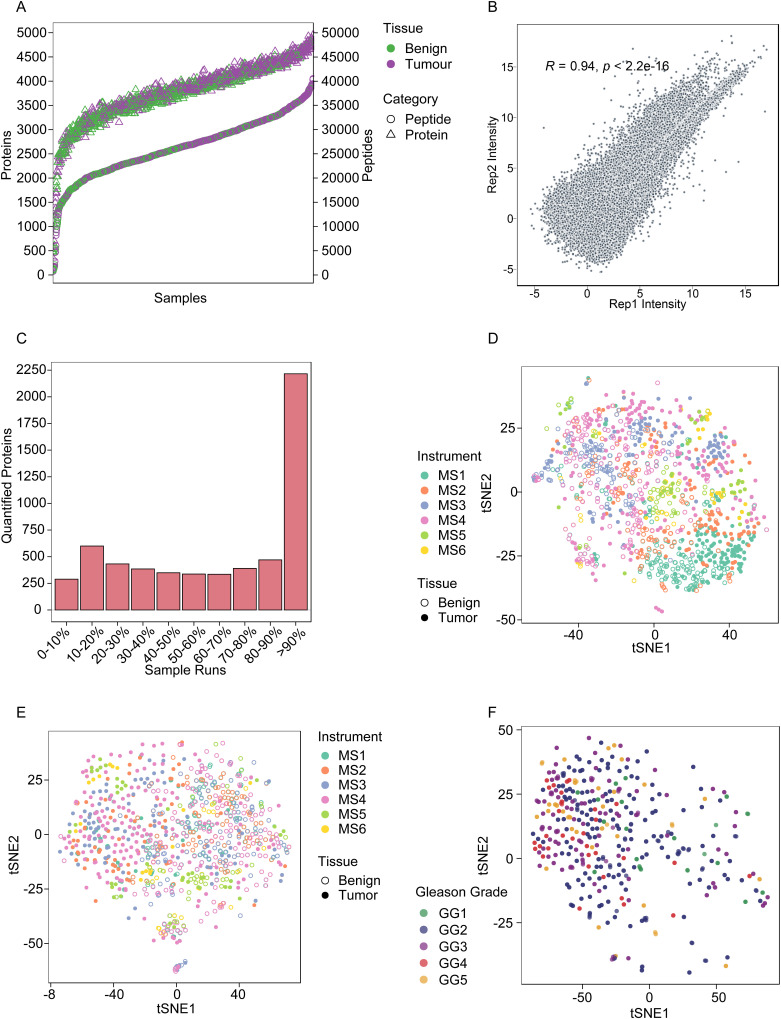
Overview of proteomic data. **(A)** Proteins and peptides quantified in tumour and benign samples (n = 1,348). A total of 53,713 peptides and 5,803 proteins are quantified. The purple colour shows the number of proteins and peptides quantified in tumour samples, and the green colour shows that in benign samples. Compared with tumour samples, a smaller number of proteins are quantified in benign samples. **(B)** Technical reproducibility of dataset shown by Pearson’s r among replicates. **(C)** Proteins quantified in different fractions of samples. ∼3,500 proteins are quantified in 50% or more samples. **(D)** tSNE projection of proteomic data before DIA-NN normalization. Samples analysed using different mass spectrometers (MS1-6) are shown with different colours. Clusters of tumour and benign samples are shown in different shapes. **(E)** tSNE projection of proteomic data after DIA-NN normalization showing no batch effects. **(F)** tSNE projection of tumour samples coloured by the GG. No grouping can be observed.

Proteomic profiles of all samples including controls were acquired by DIA-MS in technical duplicate at ProCan (36) using operating conditions that enable reproducible and high-throughput data acquisition across six SCIEX TripleTOF 6600 mass spectrometers (31, 35). We quantified 5,803 proteins (Fig S3A), with tumour samples showing a higher number of quantified proteins (average proteins per sample = 3,922) compared with benign samples (average proteins per sample = 3,587) (Fig S3A). The technical reproducibility of the cohort was evaluated by the Pearson correlation coefficient (Pearson’s r) among the sample replicates. There was a high degree of correlation between technical replicates of all samples with an average Pearson r of 0.94 (Fig S3B). Of the 5,803 proteins identified, >2,200 proteins were quantified in >90% of the samples and around 800 proteins were quantified in <20% of the samples (Fig S3C).

The t-distributed stochastic neighbour embedding (tSNE) analysis did not show batch effects from sample preparation. No batch effects from different mass spectrometers could be observed after DIA-NN normalization (see the Materials and Methods section; Fig S3D and E). The tSNE analysis also showed a clear difference between benign and tumour samples (Fig 1B). As expected, CTRL-A and CTRL-B samples are distinct from one another (Fig 1B), implicating variation from both the mass spectrometer and sample preparation (CTRL-A) and variation from the mass spectrometer alone (CTRL-B). Tumour samples of high GGs (GG4 and GG5) were only partially separated from other groups, and separation of intermediate groups (GG2 and GG3) was barely visible (Fig S3F). A heatmap of the protein matrix showed distinct expression patterns of tumour and benign samples; however, no patterns were observed for GGs, which indicates that the GG system alone does not explain the proteomic heterogeneity (Fig 1C). Tumour and benign samples were compared by differential expression analysis as a data pre-processing step (Fig 1D), resulting in the identification of 512 tumour-enriched proteins. These proteins were employed for the subsequent differential expression analysis, machine learning, and survival analysis (Fig 1E).

### Pre-processing by differential expression analysis between tumour and benign samples

**Figure S4.**
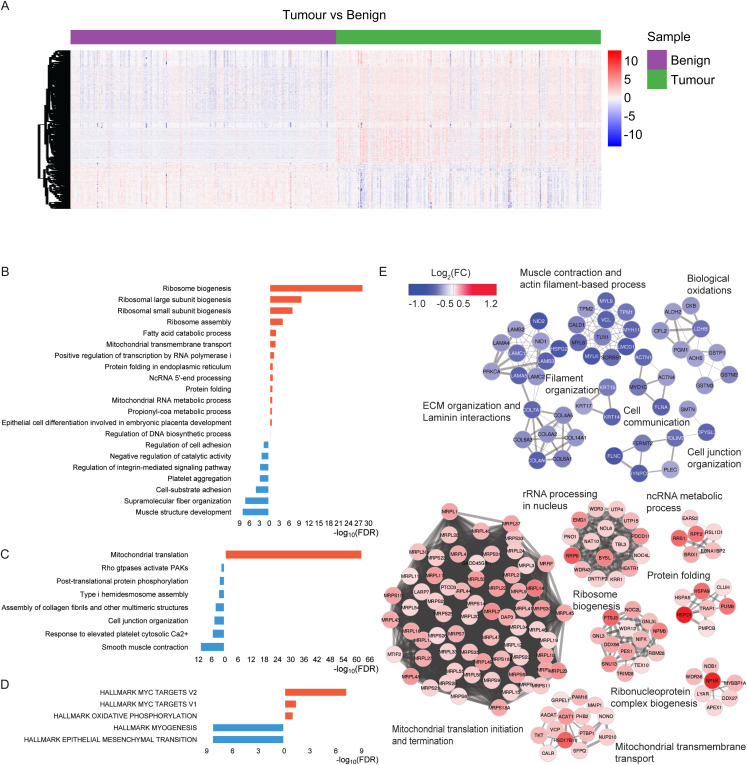
Dysregulated pathways in tumour samples. **(A)** Heatmap representation of the expression levels of differentially expressed proteins between tumour and benign samples shown in Fig 1D. Expression values are converted to z-scores. Samples are sorted according to tissue types (tumour versus benign) on the x-axis, whereas proteins are clustered on the y-axis. **(B, C, D)** Biological pathways including GO biological processes (B), Reactome pathways (C), and hallmark gene sets (D) enriched for the tumour versus benign differentially expressed proteins. Red bars indicate pathways enriched in up-regulated proteins, and blue bars indicate pathways enriched in down-regulated proteins. **(E)** PPI network components were obtained using the MCODE algorithm, showing the enriched biological processes and proteins. Up-regulated and down-regulated networks and associated proteins are coloured by FC. Up-regulated proteins are coloured in red, and down-regulated proteins, in blue. The width of the edge (between nodes) indicates the strength of the connection. A functional description is provided beside each component.

To build a protein-based prognostic signature that stratifies GG2 and GG3 patients, we selected tumour-enriched proteins by performing a differential expression analysis between tumour and benign samples. In this pre-processing step, all tumour and matched benign samples were used with the full set of 5,803 proteins. The analysis resulted in 512 tumour-enriched proteins, of which 368 were up-regulated and 144 were down-regulated in tumour samples (Fig 1D). The expression patterns of these differentially expressed proteins are shown in Fig S4A where proteins in the top cluster (up-regulated proteins) exhibited considerably higher expression in tumour samples compared with benign samples. Proteins in the bottom cluster were down-regulated in tumour samples.

Pathway enrichment analysis and protein–protein interaction (PPI) networks (38) revealed that most of the up-regulated pathways were related to ribosomal RNA processing, mitochondrial transmembrane transport, and protein folding (Fig S4B and C). When searched within the hallmark gene sets (38), the up-regulated proteins were also found to be enriched in the MYC (proto-oncogene) target V1 and V2 gene sets, which are known to be associated with tumour aggressiveness (Fig S4D).

Pathways and gene ontology (GO) processes that were significantly enriched in benign samples compared with tumour samples included muscle structure development, supramolecular fibre organization, and response to elevated platelet cytosolic Ca2+ (Fig S4B–D). Of the top 20 differentially expressed proteins identified in tumour samples, four (MDH2, FASN, EPCAM, and HSD17B10) are targetable by FDA-approved drugs, whereas two (AMACR and GLYATL1) are potentially targetable (39) and are of potential interest for future research.

The protein complexes identified in the PPI network, using the Molecular Complex Detection (MCODE) method showed up-regulation of a large set of ribosomal proteins (both large and small subunits) that promote the process of protein translation, up-regulation of proteins actively involved in the RNA metabolic process (RRS1, RPF2, BRIX1, RSL1D1), ribosome biogenesis (FTSJ3, DDX56, NPM3, GNL3, SNU13), and protein folding (HSPD1, HSPA9, HSPA5, PUM3) (Fig S4E). This is consistent with previous work showing the overexpression of proteins associated with cell adhesion, mitochondrial and ribosomal biogenesis, and translation in PCa tissue samples (40). Thus, we identified a list of differentially expressed proteins within tumour tissues for use in the downstream analyses, and identified a number of potentially important proteins and pathways in PCa.

### Stratification of GG2 and GG3 patients

**Figure 2.**
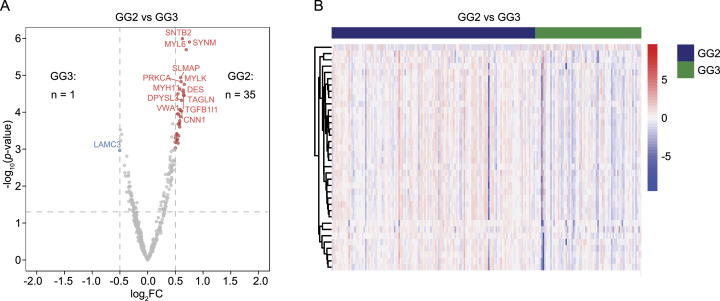
Differentially expressed proteins in the GG2 versus GG3. **(A)** Volcano plot showing the GG2 (n = 35) and GG3 (n = 1) enriched proteins in tumours. Significant proteins are presented in red and blue colours, whereas other proteins are coloured in grey. Only a small number of proteins were found to be significant using differential expression analysis, whereas most of them showed low FC. **(A, B)** Heatmap representation of the expression levels of differentially expressed proteins between GG2 and GG3 samples shown in (A). Expression data are converted to z-scores. Samples are shown on the x-axis, whereas proteins are clustered on the y-axis.

To characterize the PCa samples from the GG2 and GG3, we performed a differential expression analysis between the two GGs using the 512 tumour-enriched proteins. Of these, 35 proteins were significantly enriched in the GG2 and one protein was enriched in the GG3 (FC > 1.5 and < 0.67, *P* < 0.05; Fig 2A). The significantly differentially expressed proteins formed two clusters based on their expression in GG2 and GG3 samples (Fig 2B). As the set of significantly up- and down-regulated proteins was small, no significantly enriched pathway between the GG2 and GG3 was identified. However, of the 35 up-regulated proteins in the GG2, two (TGFB1 and FLNA) are involved in androgen receptor pathways, three (FLNC, DES, and LMOD1) have previously been associated with better prognosis in PCa (25, 41, 42, 43, 44, 45), four (PRKCA, ACTN1, AOC3, and LDHB) are targets for FDA-approved drugs (39), and three (MYLK, FLNA, and FLNC) are potential drug targets (39). The results suggested likely biological differences between the GG2 and GG3 and identified several potential diagnostic and prognostic biomarkers that could be further investigated.

**Figure 3.**
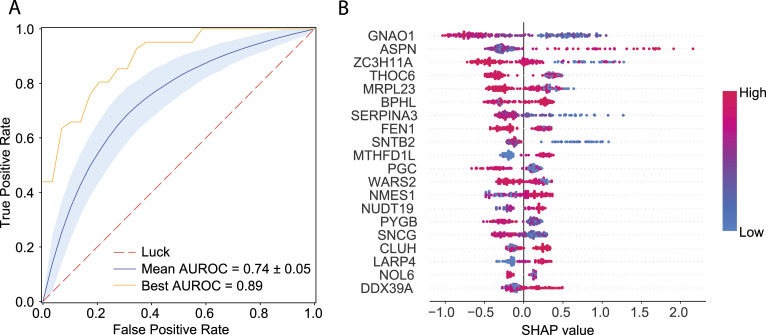
Machine learning of the GG2 versus GG3. **(A)** ROC curves for the best and average models for predicting GG2 and GG3 samples based on 1,000 Monte Carlo runs by XGBoost. The red dashed line represents the random guess, the blue solid curve shows the mean ROC curve over 1,000 Monte Carlo runs, the blue band represents one SD of the curves, and the orange curve shows the best ROC curve. **(B)** SHAP values of the top 20 most significant proteins to distinguish between GG2 and GG3 samples, sorted (from top to bottom) by their respective absolute mean SHAP values. SHAP values of proteins in different samples are shown on the horizontal axis; the top 20 proteins are sorted (by importance) from top to bottom on the y-axis. The colours from blue to red indicate protein expression levels from low to high. The vertical zero line (SHAP value = 0) is the line that has no impact on prediction, whereas the values on the left and right sides represent negative and positive impacts on prediction.

To stratify GG2 against GG3 patients by machine learning, a dataset containing only GG2 and GG3 patients and the 512 tumour-enriched proteins was used. The results, aggregated over 1,000 Monte Carlo cross-validation runs of an XGBoost classifier with 80% training and 20% testing splits, demonstrate that the difference between the GG2 and GG3 can be predicted from protein intensities with high accuracy (Fig 3A). The receiver operating characteristic (ROC) curve of the best model had an area under the ROC (AUROC) of 0.89, with a mean AUROC of 0.74 (Fig 3A). To obtain a reproducible list of the top 20 most significant proteins in separating GG2 and GG3 samples, SHapley Additive exPlanations (SHAP) values were calculated over the entire cohort (Fig 3B; see the Materials and Methods section).

**Figure 4.**
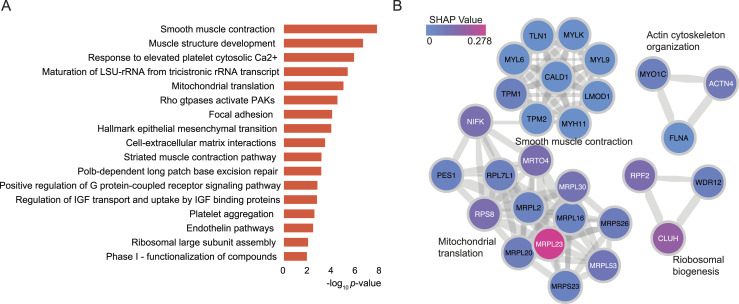
Differentially expressed proteins and pathways in GG2 and GG3 PCa. **(A)** GO biological processes, Reactome pathways, and hallmark gene sets enriched for the selected significant proteins. **(B)** PPI network components obtained using the MCODE algorithm, showing the enriched biological processes and proteins. Proteins are coloured according to the absolute mean SHAP values. The width of the edge (between nodes) indicates the strength of the connection. A functional description is provided beside each component.

To study the dysregulated biological pathways in the GG2 and GG3, a total of 127 proteins were selected by taking the union of 36 differentially expressed proteins (Fig 2A) and the top 100 proteins from the machine learning that contains the top 20 proteins in Fig 3B. Pathway enrichment analysis and PPIs from Reactome pathways (38) for these proteins highlighted an overrepresentation of proteins involved in muscle structure, ECM organization, and response to elevated platelet cytosolic Ca2+ pathways (Fig 4A). When compared against the hallmark gene sets, enrichment for proteins in the epithelial–mesenchymal transition gene sets was observed (38) (Fig 4A). The significant protein complexes identified in the PPI network using MCODE (Fig 4B) included proteins involved in smooth muscle contraction (CALD1, TLN1, TPM2, TPM1, four myosin proteins), actin cytoskeleton proteins (ACTN4, MYO1C, FLNA), and mitochondrial translation (ribosomal subunit proteins). Most of these PPI proteins had high mean importance scores when GG2 samples were compared to GG3 samples. Our findings extend upon previous research showing that CALD1, TPM2, and TPM1 can be used as potential diagnostic biomarkers for PCa (46). Although these findings are of biological interest, further modelling is required to better understand the biological pathways associated with each GG, and thus improve the GG prognostic performance.

### Protein-based prognostic signature for BCR

**Figure 5.**
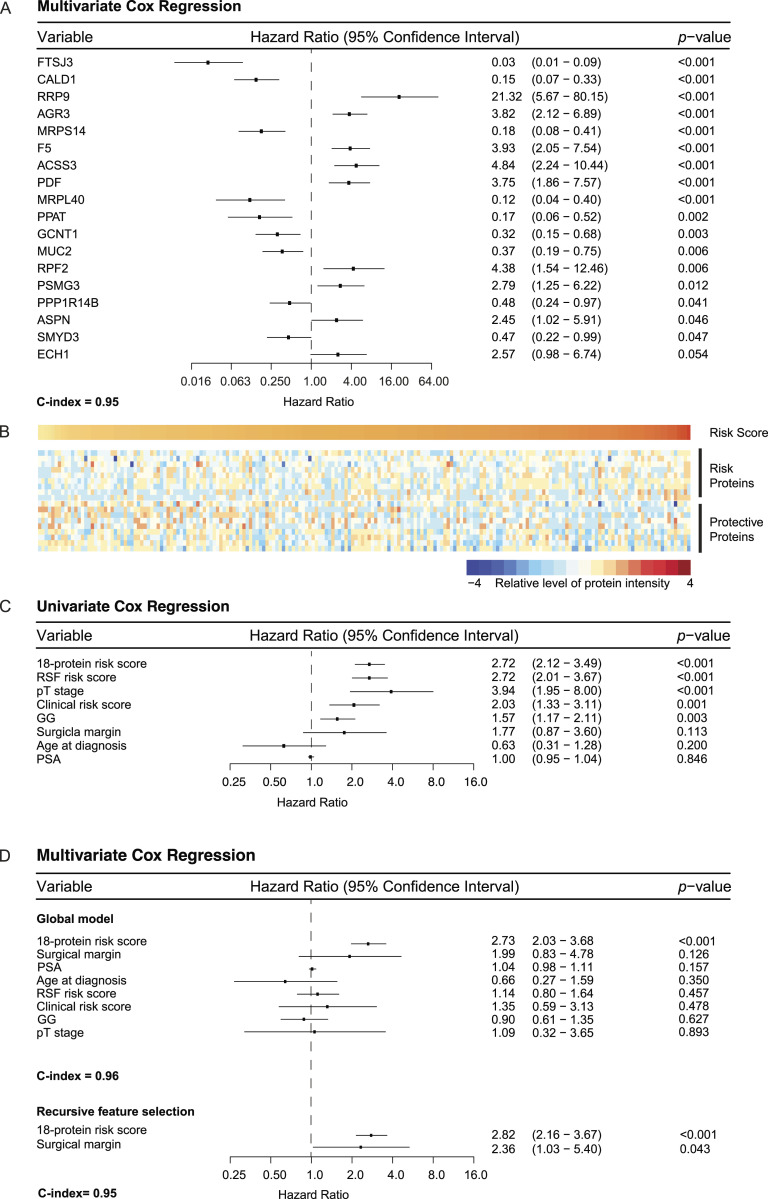
Survival analysis of BCR-free survival (BCRFS) of PCa. **(A)** Forest plot showing the 18 proteins with their individual hazard ratios, *P*-values, 95% CIs, and C-index of the final multivariate Cox model. **(B)** Heatmap showing protein intensities sorted by a risk score and clustered for each of the two groups: risk and protective proteins. The column denotes patients, and the row indicates the 18 proteins. PCa samples with positive regression coefficients expressed risk proteins, whereas samples with negative regression coefficients expressed protective proteins. **(C)** Forest plot comparing the importance of the 18-protein signature with RSF-based risk score and with other clinical variables using univariate Cox models: pT stage (pT1 versus pT2), surgical margin (positive versus negative), and age at diagnosis (<64 versus ≥64). **(D)** Forest plot showing a simple multivariate Cox model that includes the 18-protein signature, RSF-based risk score, and other clinical variables. With recursive feature selection, the 18-protein signature remains the most important variable, with a stable C-index (from 0.96 to 0.95).

To overcome one of the limitations of the GG system, exemplified in an inability to differentiate prognosis between the GG2 and GG3 in our dataset (Fig S2), a protein-based signature was constructed. First, 100 runs of multivariate Cox regression (47) with least absolute shrinkage and selection operator (LASSO) regularization were performed on the 512 tumour-enriched proteins using 20-fold cross-validation (see the Materials and Methods section). For each run, a list of significant proteins was obtained, and a merged list of these proteins was collated and ranked according to the descending order of mean significance of individual proteins over all the 100 runs. A subset comprising the top 25 of these proteins was then used to model multivariate Cox regression with recursive feature selection, yielding a final list of 18 proteins (Fig 5A). Almost all of the 18 proteins were significantly associated with BCR with a concordance index (C-index) (48) of 0.95 (Fig 5A), indicating robust prognostic power from these proteins.

A patient’s risk score was calculated as the sum of the intensities of each of the 18 proteins, multiplied by the corresponding regression coefficients (Fig 5B and see the Materials and Methods section). The midpoint of risk scores was used as the threshold to dichotomize patients into either a high-risk or a low-risk group. This two-step process gave rise to an 18-protein signature. To assess the prognostic power, the 18-protein signature was benchmarked with another signature calculated from the top 20 proteins identified by a random survival forests (RSF) (49) model and with other clinicopathologic variables including the GG, clinical risk, PSA, surgical margin, age at diagnosis, and pathological T stage (pT stage). The 18-protein signature showed the strongest association with BCR among all variables in the univariate Cox regression analysis (Fig 5C). This was also true in a multivariate Cox regression analysis after adjusting for the clinicopathologic variables and the 20-protein RSF signature independent of recursive feature selection (Fig 5D). This confirms that the 18-protein signature is not confounded by other clinicopathologic variables and can be considered an independent prognostic factor. The stable concordance index of all these models further suggests that the 18-protein signature can explain most of the association with BCR.

**Figure S5.**
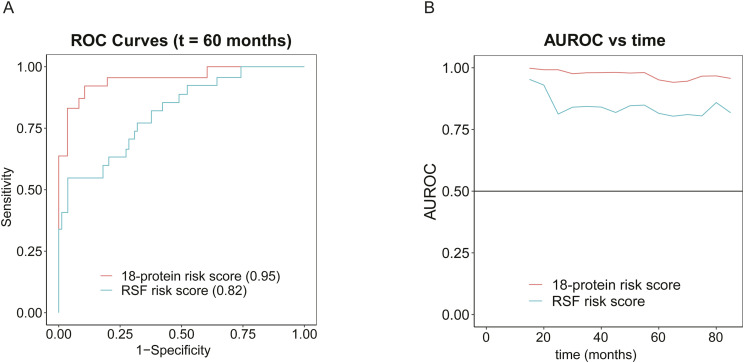
Survival analysis of BCRFS. **(A)** ROC curves with respective AUROCs at a 5-yr (60 mo) follow-up for the 18-protein risk score and RSF-based risk score. **(B)** Time-dependent AUROCs of the 18-protein risk score and RSF-based risk score.

Moreover, our 18-protein signature was compared with the 20-protein RSF signature using a time-dependent ROC analysis, which measures how well an independent variable can differentiate between target classes at different time points in the study. The comparison of time-dependent ROC curves after 60 mo for both risk scores showed an AUROC of 0.95 for the 18-protein signature and an AUROC of 0.82 for the RSF signature (Fig S5A). Further comparison demonstrated the higher predictive power of the 18-protein signature over time compared with the RSF signature (Fig S5B). RSF uses bootstrapped samples in each tree to avoid overfitting and generalizes well on unseen test datasets (49). For this reason, it is noteworthy that our 18-protein signature outperformed the RSF signature even in the absence of a validation dataset.

**Figure 6.**
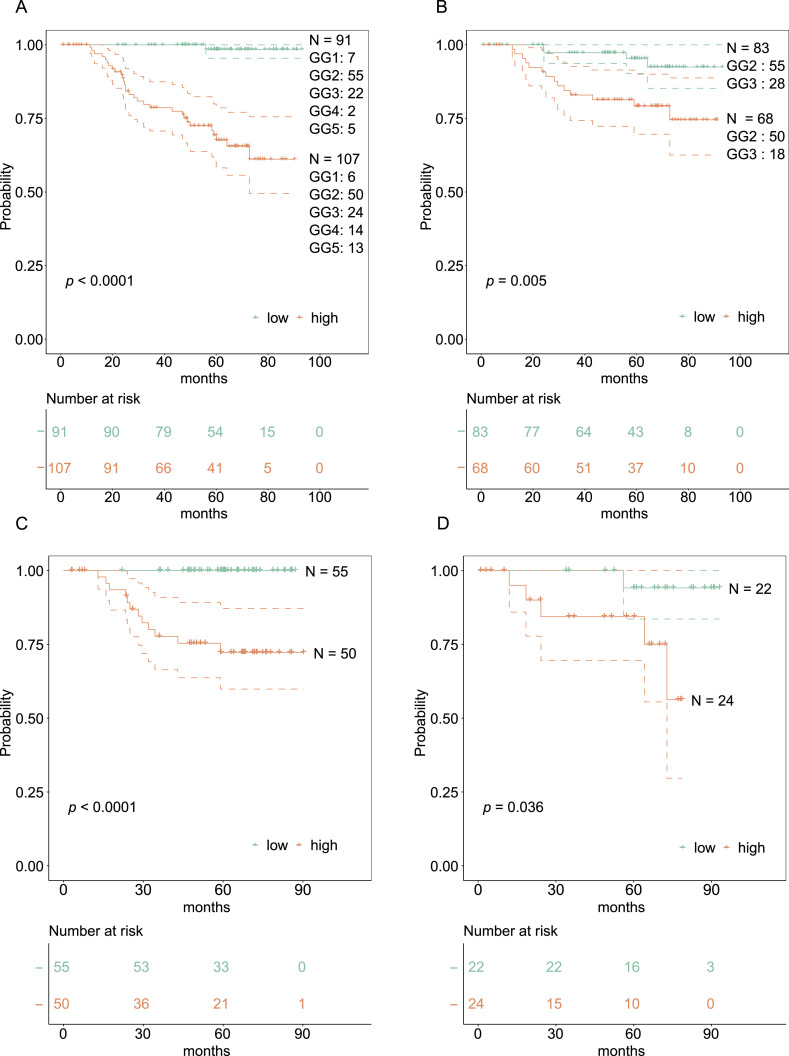
Kaplan–Meier (KM) curves for BCRFS. KM curves with 95% CIs of the low- and high-risk groups based on the 18-protein risk score, along with respective numbers of samples corresponding to each GG. Vertical lines illustrate patients who were censored at the time of their last clinical follow-up visit. The *P*-value shows the significance of the difference between survival estimates evaluated by the log-rank test. Coloured values represent the number of patients in each group under risk. **(A)** KM curves for PCa patients in all GGs. **(B)** KM curves for PCa patients in the GG2 and GG3. **(C)** KM curves for PCa patients in the GG2 only. **(D)** KM curves for PCa patients in the GG3 only.

The dichotomized Kaplan–Meier curve with the low *P*-value (<0.0001) indicated substantial predictive power by the 18-protein signature (Fig 6A). Overall, there were more patients with the GG2 and GG3 in our cohort compared with the GG1, GG4, and GG5. Interestingly, the number of patients with the GG2 and GG3 was equally distributed between the low-risk and high-risk groups (GG2: 55 and 50; GG3: 22 and 24, respectively), indicating that our protein-based signature is independent of the GG. To confirm this, we applied the 18-protein signature within the group of patients including both the GG2 and GG3 (Fig 6B), with the GG2 only (Fig 6C), and with the GG3 only (Fig 6D). The 18-protein signature was able to identify a subgroup of patients with a higher risk of developing BCR within each GG, confirming its independence of the GG, and suggesting potential clinical utility.

**Figure S6.**
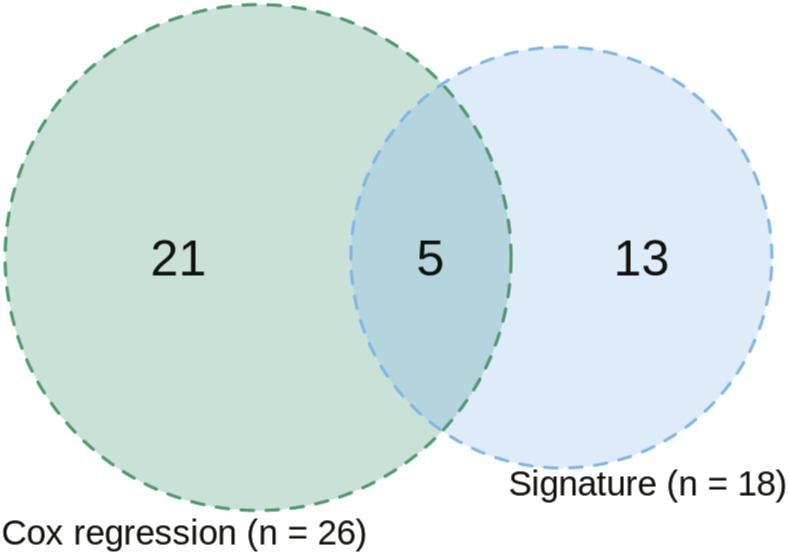
Set of unique proteins. A set of 39 unique proteins was extracted by taking the union of 18 signature proteins and 26 proteins from a univariate Cox regression model. Five proteins were found overlapping between the two sets.

**Figure 7.**
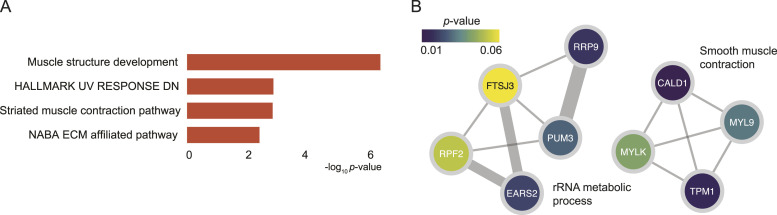
Significant biological pathways identified from a univariate Cox regression model. **(A)** GO biological processes, Reactome pathways, and hallmark gene sets enriched for the selected significant proteins. **(B)** PPI network components obtained using the MCODE algorithm, showing the enriched biological processes and proteins. Proteins are coloured according to the *P*-values from the BCRFS analysis. The width of the edge (between nodes) indicates the strength of the connection. A functional description is provided next to each component.

By taking the union of the 18 signature proteins (Fig 5A) and 26 proteins that were significantly associated with BCR in a univariate Cox regression model (*P* < 0.05), a total of 39 unique proteins (Table S2 and Fig S6) were analysed to study the association between biological pathways and BCR. Among these 39 proteins, five (F5, CALD1, RRP9, MUC2, and AGR3) were identified in common (see the Materials and Methods section), and six were related to androgen-regulated genes (F5, CALD1, TPM1, PUM3, ANXA4, and MYLK) (50). Most of the 18 signature proteins were not involved in common biological pathways and thus contribute unique biological information. However, when including all 39 proteins, several enriched pathways were identified. This included muscle structure development (CALD1, MYL9, MYLK, TPM1) and rRNA metabolic processes (RRP9, PUM3, EARS2, RPF2, FTSJ3) (Fig 7A and B). Of the total 39 proteins, two (F5 and ANXA4) (39) are targetable by FDA-approved drugs and three (TMEM126B, EARS2, and MYLK) are potentially targetable (39). Among the list of 26 proteins associated with BCR in the univariate Cox regression modelling, F5 (HR 1.7, 95% CI [1.2, 2.4]), TMEM126B (HR 1.5, 95% CI [1.1, 2.0]), and EARS2 (HR 1.9, 95% CI [1.1, 3.2]) were associated with an increased risk of BCR, suggesting potential utility for further investigation as drug targets in clinical practice.

Table S2. Summary of 39 Signature Proteins.

## Discussion

We performed a large-scale quantitative proteomic analysis from 278 PCa patients with primary tumour and matched benign tissue samples, each analysed in technical duplicate. We identified differentially expressed proteins and multiple signalling pathways related to PCa development and progression. In addition, we built an 18-protein signature that overcomes the limitations of the GG in distinguishing between intermediate-risk PCa patients and that has a higher prognostic value compared with the standard classification. We were also able to identify potential therapeutic targets that can be explored for their utility in the treatment of PCa. The main finding of this study is that patients with GG2 adenocarcinomas of the prostate (clinically the most common subgroup) could be significantly and independently divided into two subgroups with a differential risk of BCR by our proteomic-based survival analysis, albeit an exploratory investigation.

The pathway enrichment analyses on tumour-enriched proteins showed that pathways were related to protein folding, rRNA processing, ECM organization, mitochondrial translation initiation, and PCa development. Among the top 20 differentially expressed proteins, several proteins (AMACR, MDH2, FASN, HSD17B10) were involved in metabolic-related pathways (51, 52, 53). Although few proteins were related to androgen (HSD17B10, F5, PUM3) (54) and DNA damage repair (NPM1, FEN1) (54) pathways, 16% of our 512 differentially expressed proteins overlapped with the overexpressed genes in PCa (55). In addition, AMACR, FASN, IGFBP2, and PHB identified in our analysis are among biomarkers previously suggested for PCa diagnosis (40).

Four of the top 20 differentially expressed proteins (MDH2, FASN, EPCAM, and HSD17B10) are targetable with FDA-approved drugs, whereas two are potentially targetable proteins (AMACR and GLYATL1) (39). AMACR was the top significantly up-regulated protein in the tumour tissue. AMACR has a major role in fatty acid oxidation and has previously been found to be overexpressed in PCa at the proteomic and transcriptomic levels, confirming its validity as a potential biomarker (56, 57, 58, 59). Among the four FDA-approved targetable proteins, MDH2 is known to be overexpressed in PCa and castrate-resistant PCa, highlighting its role in PCa progression (23) and resistance to chemotherapy (60). FASN is a key enzyme in de novo fatty acid synthesis and has been found to be overexpressed in castrate-resistant PCa and many other types of solid tumours (51). It is also associated with PCa progression, mainly through the activation of the PI3K/Akt/mTORC1 pathway, with a recent study suggesting the potential therapeutic benefit of its inhibition to overcome resistance to anti-androgen treatment (52). EPCAM is a marker for cancer stem cells that are associated with cancer proliferation, adhesion, and differentiation, and it is overexpressed in different types of cancer, including PCa (61). In a meta-analysis, EPCAM overexpression was associated with a higher risk of BCR and the development of bone metastasis (62). Finally, HSD17B10 is involved in different metabolic pathways, has an important role in regulating tissue androgen levels, and may be involved in PCa progression through androgen-independent pathways (53). Further studies will be required to confirm the value of these potential therapeutic targets in PCa management.

Our analyses identified 39 proteins significantly associated with BCR, of which five were listed in the Human Protein Atlas database (63) either as FDA-approved targetable proteins (F5 and ANXA4) or as potentially targetable proteins (TMEM126B, EARS2, and MYLK) (39). None of these proteins overlapped with a published list of potential biomarkers for PCa aggressiveness or treatment resistance (56). This may be due to the nature of our study cohort being a treatment-naïve patient population that was not yet exposed to anti-androgen treatment. However, three proteins (HNRNPA2B1, MRPS22, and PUM3) from our analysis were identified within The Cancer Genome Atlas (TCGA) list of genes associated with poor prognosis (64). Our results suggest the potential usefulness of F5, TMEM126B, and EARS2 as potential therapeutic targets. Using PPI network analysis and tissue-specific gene co-expression network analysis, F5 was identified as one of the core genes in PCa (65). Interestingly, F5 was also associated with an increased risk of breast cancer and the activation of the immune microenvironment (66). TMEM126B is a complex I assembly factor that is critical for oxidative stress and inflammatory response (67). Previous studies have demonstrated its role in response to chronic hypoxia through HIF-1–dependent mechanisms (68). Although the role of TMEM126B in PCa is not fully explored, its interaction with HIF-1–dependent pathways, which play a critical role in PCa progression (69, 70), warrants further exploration. EARS2 is involved in mitochondrial protein synthesis and was found to be associated with breast, pancreatic, renal, and colorectal cancers (71, 72). There is some evidence of the co-expression of EARS2 with PALB2 in breast and pancreatic cancer and the association of their overexpression with poorer outcomes (72). This finding suggests that PALB2 may also be involved in PCa progression and response to treatment (73, 74, 75).

Despite the established prognostic value of the GG system and its use in PCa management, its limitations are well-recognized (8, 9, 12). Previous studies have illustrated the differences between the GG2 and GG3 on the metabolomic level, with a higher intensity of phosphatidylcholines, and cardiolipins, among others, within GG3 samples, suggesting the involvement of the differential biological pathway (17). Similarly, Kawahara et al performed proteomic analysis on 50 PCa tissue samples and identified a panel of 11 proteins that were associated with high-grade (GG4 and GG5) versus low-grade (GG1 and GG2) PCa (25). Interestingly, this 11-protein panel was not able to distinguish samples within the GG3 (25). In another study, a five-gene signature was constructed using data from the GEO and TCGA datasets, which was independent of the Gleason score when dichotomized as less or more than 7 (76). However, the prognostic power of this signature was not explored within each GG (especially the intermediate groups, GG2 and GG3).

In our analysis, there was an overlap between the GG2 and GG3 in terms of their risk of developing BCR (Fig S2), reflecting the limitations of GG stratification. Our study identified 35 up-regulated proteins in the GG2 compared with the GG3. These proteins were related to muscle structure development, epithelial-to-mesenchymal transition, metabolic pathways, and ECM interaction. As expected, most up-regulated proteins are related to cancer genes (39), with seven of them known to be enhanced in PCa (SYNM, DES, MYH11, TAGLN, CNN1, LMOD1, and PGM5) (39). Four up-regulated proteins within the GG2 are FDA-approved drug targets (PRKCA, ACTN1, AOC3, and LDHB) (39), and three are potential drug targets (MYLK, FLNA, and FLNC) (39). In addition, several proteins that were up-regulated in the GG2 can be used as potential prognostic biomarkers that need further investigation. Of these, FLNC, a potential drug target that is involved in cell–extracellular matrix interaction, has been associated with progression-free survival and a lower risk of BCR (41, 42). DES, a cancer-enhanced gene that is involved in Aurora B signalling and striated muscle contraction, has been found to be underexpressed in PCa and is associated with better prognosis (43, 44, 45). Finally, LMOD1, a PCa-enhanced gene, has lower expression in high-grade and metastatic PCa (25). Further research is required to determine the utility of those proteins as prognostic biomarkers at the time of PCa diagnosis.

To overcome the limitations of the GG, we have built a protein-based signature and explored its prognostic power together with and in comparison with the GG. Our 18-protein signature identified patients at a higher risk of developing BCR with high accuracy. Its prognosis was maintained even after adjusting for other clinical variables, including the GG, pT stage, and baseline PSA. In addition, the 18-protein signature was independent of the GG, being able to identify patients at a higher risk of developing BCR within each of the GG2 and GG3 separately. This distinction is of considerable clinical importance, considering the recent BCR management guidelines, which depend only on the GG and PSA doubling time (77). Further exploration of this protein-based signature for patients planned for active surveillance would be useful considering its potential ability to identify patients at a higher risk of progression independent of their clinical risk score (PSA, GG, and pT stage) (3). Our results both complement and extend upon recent proteomic studies in PCa (27). First, the novel contribution of our work is to present a substantially larger cohort size (n = 278) than previous studies, which typically comprise <100 patients (27). Second, our study is able to identify potential novel therapeutic targets and build a prognostic signature that is completely independent of the GG, with the ability to identify patients at a higher risk of developing BCR within the relatively indolent GG2.

Although BCR is a problematic endpoint (78), evidence suggests that patients who develop BCR are at a higher risk of developing clinical progression (79). The incidence of BCR after radical prostatectomy can reach up to 40% (79), and it is significantly associated with clinical recurrence, metastasis, and cancer-specific death (80). Consequently, predicting a BCR risk using various clinical indicators to guide clinical work-up is classically based on ISUP grouping, PSA at diagnosis, clinical stage, etc. The effectiveness of combining radiological and clinical parameters to measure the BCR risk was also evaluated, and it was shown to increase the predictive accuracy of the risk stratification method (81). It will be important to further investigate and validate the utility of our 18-protein signature on selecting the group of patients at a higher risk of clinical progression and poorer survival. Finally, our dataset will serve as an important public resource for the scientific community seeking to understand the proteomic landscape in PCa.

This study is hampered by the unavailability of metastatic relapse and mortality data and the smaller number of patients within the GG1, GG4, and GG5, which prevented us from confirming the prognostic value of the 18-protein signature within these GGs. In addition, it remains to be determined to what extent this signature will be transferrable to other proteomic platforms and whether it can be detected reliably by semi-quantitative techniques such as multiplexed immunohistochemistry. Although we did not have access to a validation cohort to verify our findings at the time of these analyses, our data will become an important resource for any future work requiring a validation dataset. Showing that our 18-protein signature had higher significance and AUROC as compared to the 20-protein RSF signature does provide a level of confirmation because the RSF model works on selecting bootstrapped samples in each tree while computing the importance of proteins. This process mimics internal cross-validation, avoids overfitting, and has been shown to generalize well on future data (49).

We conclude that PCa proteomic analysis is a promising tool for understanding the biological pathways associated with PCa development and progression. Our analysis has identified several novel therapeutic targets, and possible diagnostic and prognostic biomarkers that can be further investigated in pre-clinical and clinical studies. Importantly, we have also built an 18-protein signature that was predictive of BCR and is independent of the GG. Further work is required to first validate our findings in an independent cohort and then to integrate them into clinical practice.

## Materials and Methods

### Biospecimen collection and pathology and clinical data

**Figure S7.**
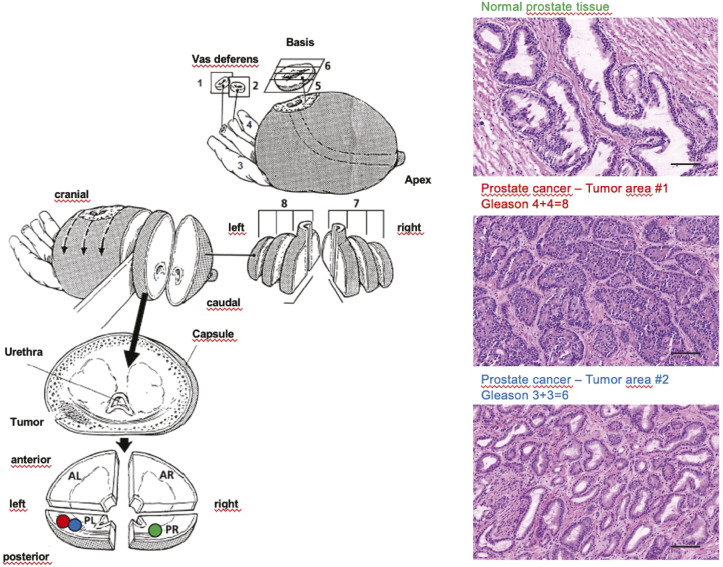
Fresh-frozen prostate tissue. Biobanking of fresh-frozen prostate tissue from radical prostatectomy specimens (left; adapted with permission from Springer Nature; RH Hruban et al, Surgical Pathology Dissection, Springer Science + Business Media New York 1996) and histological evaluation of cancer and non-cancerous tissue using haematoxylin–eosin staining of fresh-frozen tissue sections (right). In detail, tissue samples (prostate cancer, benign prostatic hyperplasia) were collected as part of the ProCOC, a prospective biobank study that has now collected tumour (mainly acinar adenocarcinoma of the prostate) and non-tumour (mainly benign hyperplasia of the prostate) tissues from more than 1,000 radical prostatectomies. All patients with localized PCa scheduled for radical prostatectomy in curative intent at the University Hospital Zürich were asked to participate in the ProCOC biorepository ahead of surgery since 2008. The use of fresh-frozen tissues of the ProCOC cohort has already been reported in previous studies. The native radical prostatectomy specimens were processed in the Department of Pathology and Molecular Pathology, University Hospital Zürich, immediately after surgery. A complete cross section of each fresh, unfixed, and cooled (4°C) prostate specimen was collected from each prostatectomy specimen and divided into four quadrants; that is, the first slice after dissection of the apex was quartered and snap-frozen in four separate blocks, using a special procedure to ensure ideal sample quality. Only samples with sufficient tumour tissue (index tumour >5 mm diameter) were selected for subsequent proteomic analysis. All fresh-frozen samples were stored at −80°C. After formalin fixation overnight, the rest of the specimen was processed accordingly. The central tissue biobank of the University Hospital Zürich is specifically accredited for this purpose by the Swiss Biobanking Platform. Haematoxylin-and-eosin–stained sections of the four frozen blocks were sliced for immediate evaluation regarding tumour load and margins in synopsis with the standard formalin-fixed, paraffin-embedded histology. The size of a single fresh-frozen tissue punch was ∼1 mm3 (diameter, 1 mm; length, 1–2 mm; wet weight, ∼800 μg). Clinical and histological data: follow-up information regarding persistence of PSA or its re-emergence as biochemical relapse, and disease-specific and non–disease-specific cases of death were recorded from the electronic health records, or whether patients had their postoperative follow-up outside of our care centre at least bi-annually by contacting patients directly and their caring physicians. Patients who did not achieve a PSA nadir were excluded from the analysis. Scale bar = 100 μm.

The sample collection of this study was approved by the Cantonal Ethics Committee of Zürich (KEK-ZH-No. 2008-0040). Detailed information on the patients and samples is provided in Table S1 and Fig S7.

### Sample preparation and mass spectrometric acquisition

About 0.5 mg of fresh-frozen prostate tissue was punched from the sample, weighed, and processed for each biological replicate via the workflow as described in Fig S7.

An Eksigent nanoLC 425 HPLC system operating in a microflow mode, coupled online to a TripleTOF 6600 system (SCIEX), was used for the analyses. The peptide digests (2 μg) were injected onto a C18 trap column (SGE TRAPCOL C18 G 300 μm × 100 mm) and desalted for 5 min at 8 μl/min with solvent A (0.1% [vol/vol] formic acid). The trap column was switched in line with a reversed-phase capillary column (SGE C18 G 250 mm × 300 μm ID 3 μm 200 Å), maintained at a temperature of 40°C. The flow rate was 5 μl/min. The gradient started at 2% solvent B (99.9% [vol/vol] acetonitrile, 0.1% [vol/vol] formic acid) and increased to 35% over 69 min. This was followed by an increase of solvent B to 95% over 4 min. The column was washed with 95% solvent B for 5 min, then decreased to 2% solvent B over 3 min followed by a 13-min column equilibration step with 98% solvent A. For SWATH acquisition, peptide spectra were analysed using the TripleTOF 6600 system (SCIEX), equipped with a DuoSpray source and 50-μm internal diameter electrode and controlled by Analyst 1.7.1 software. The following parameters were used: 5500 V ion spray voltage; 25 nitrogen curtain gas; 100°C TEM, 20 source gas 1, 20 source gas 2 with 100 variable windows, as per a SCIEX technical note (Supplemental Data 1). The parameters were set as follows: lower m/z limit, 350; upper m/z limit, 1250; acquisition time, 150 ms; and window overlap (Da), 1.0; CES was set at 5 for the smaller windows, then 8 for larger windows, and 10 for the largest windows. MS2 spectra were collected in the range of m/z 100 to 2,000 for 30 ms in a high-resolution mode, and the resulting total cycle time was 3.2 s.

Supplemental Data 1. SCIEX technical note.

### Proteomic data analysis

We analysed 278 of 290 PCa patients whose malignant tissue samples were classified by pathologists alongside the matched benign tissue. A total of 12 patients were removed after QC. The entire cohort was then divided into 31 batches, with each containing between 15 and 29 samples including two control samples (CTRL-A, n = 62; and CTRL-B, n = 62) for QC and the evaluation of reproducibility (Fig S1). The samples were analysed in technical duplicate in different mass spectrometers in ProCan (31, 36).

From each patient, a malignant tissue sample and its matched benign sample were processed using the pressure cycling technology (82) in technical duplicate, and randomly selected samples were processed with both biological replicates and technical replicates. The samples were processed in 31 batches, each containing a reference sample (CTRL-A) of a homogeneous PCa tissue sample that could account for technical variation introduced during the entire pressure cycling technology–SWATH-MS sample processing methodology, and a reference sample of a homogeneous prostate tissue digest (CTRL-B) that could account for technical variation introduced during SWATH-MS.

#### DIA-based spectral library generation

DIA-MS data in wiff file format were collected for 1,475 runs and were processed using DIA-NN (version 1.8) (83). A spectral library was generated using 1,475 DIA-MS runs and consisted of 9,230 proteins and 89,408 peptides. The spectral library was used to search the complete cohort of 1,475 runs.

#### Data extraction

DIA-NN was implemented using RT-dependent normalization and with parameters given below:

-report-lib-info --out step3-out.tsv --qvalue 0.01 --pg-level 1 --mass-acc-ms1 40 --mass-acc 40 --window 9 --int-removal 1 --matrices --temp. --smart-profiling --peak-center.

Data were then filtered to retain only precursors from proteotypic peptides with Global.Q.Value ≤ 0.01. Proteins were then quantified using MaxLFQ, with default parameters (84), and implemented using the DIA-NN R package (https://github.com/vdemichev/diann-rpackage). Data were then log_2_-transformed. There were 1,475 mass spectrometry runs with 669 benign and 679 tumour samples. For downstream analysis, a final protein matrix with only benign and tumour samples (n = 1,348 samples) was used. The protein matrix showed an average of 35% missingness per individual sample. Missing values in this dataset were then imputed with a constant lower than the minimum value of the whole protein matrix to maintain the distinction between missing values and protein intensities. Sample replicates were merged. The imputed protein matrix was z-score–standardized and was then used as input for further analyses.

#### Batch effect analysis

The tSNE analysis of the data was performed on the final protein data matrix with 5,803 proteins. The instrument batch effect was observed as samples were run on six different mass spectrometers. The tSNE-based two-dimensional visualization of protein data showed that the instrument batch effect was corrected after the built-in normalization method in the software suite DIA-NN (Fig S3D and E).

### Differential proteomic analysis

Differential expression analysis between tumour and benign samples was performed on all 5,803 proteins, and analysis between GG2 and GG3 samples was performed on 512 tumour-enriched proteins. Empirical Bayes moderated t-statistics, packaged in the Limma R package, version 3.54.1, was performed to compute the *P*-value of the protein intensity between the two classes. Tumour-specific significantly expressed proteins were selected at the Benjamini–Hochberg (BH)-adjusted *P* < 0.01 and with log fold change (FC) (expressed as the difference in the group means) cut-off of ±0.5 (FC > 1.5 and < 0.67), whereas the GG2- and GG3-specific differentially expressed proteins were selected at *P* < 0.05 and with log FC (expressed as the difference in the group means) cut-off of ±0.5 (FC > 1.5 and < 0.67). Heatmaps were generated using the R package pheatmap, version 1.0.12. The complete linkage clustering algorithm was used along with the Euclidean distance as the distance measure.

### Survival analysis

#### Finding a proteomic signature

The protein dataset, containing the 512 tumour-enriched proteins, was used as the input for the survival analysis. To reduce the number of important proteins, 100 runs of multivariate Cox regression with LASSO regularization were executed on the whole dataset. The LASSO regularization hyperparameter in each run was tuned using 20-fold cross-validation. Each run returned a list of proteins with non-zero coefficients. These lists were then combined into a list of unique proteins, which was then ranked according to the mean importance of the individual proteins (average absolute coefficient over 100 runs) in descending order. The top 25 of these proteins were then used in a multivariate Cox model with recursive feature selection, which yielded the final 18 proteins. These 18 proteins were then used to construct the proteomic risk score (*S*_*j*_), for the *j*_th_ patient, as below:

$$S_{j}=\sum_{i=1}^{n}\left(\beta_{i}X_{ji}\;\right),$$

where *n* is the total number of proteins; *β*_*i*_ is the coefficient of the *i*_th_ protein; and *X*_*ji*_ is the intensity of the *i*_th_ protein, in the *j*_th_ patient.

#### Analysing performance of the proteomic risk score

The performance of the risk score was analysed in multiple ways. First, patients were dichotomized into low- and high-risk groups using the midpoint of the risk scores as a threshold, and their Kaplan–Meier (KM) curves were then plotted. Differences between survival estimates were evaluated by the log-rank test, and *P*-values were reported. The number of samples corresponding to each GG falling in both low- and high-risk groups was counted to analyse how well the KM curves justified categorization based on the GG. Furthermore, to check its performance in GG2 and GG3 patients, KM curves for the dichotomized risk score were plotted in both combined and separate GG2 and GG3 patients.

The C-index is a measure of rank correlation between the predicted risk score and the observed time points. For instance, if the predicted risk score of a sample is higher than that of another, and the observed time point of that sample is earlier than that of the other sample, then the predictions and observations are said to be concordant.

### Functional enrichment analysis

Functional and pathway enrichment analyses of significantly expressed proteins were performed using Metascape (38) along with the entire set of 5,803 proteins as the background gene set. The gene ontology (GO) biological processes, Reactome pathways, and hallmark gene sets enriched in dysregulated proteins were acquired. The input parameters were *P* < 0.05, minimum gene count of 3, and enrichment factor > 1. The *P*-values are calculated based on accumulative hypergeometric distribution and are adjusted using the BH correction. For tumour versus benign comparisons, statistically significant enriched terms were selected at an adjusted *P*-value (*q*-value or FDR) of 0.05 (−log_10_ FDR > 1.3), whereas for GG2 versus GG3 comparisons, statistically significant enriched terms were selected at a *P*-value of 0.05 (−log_10_ *P* > 1.3).

### PPI enrichment analysis

PPI enrichment analysis was performed using the Metascape (38) by incorporating the data from STRING and BioGRID databases. As a result, a network of subsets of proteins is formed where proteins in the input list form physical interaction with at least one other member in the list. To identify the functional protein complexes for the differentially expressed proteins, the MCODE algorithm was applied within the Metascape (38). MCODE detects and generates the significant protein complexes (*P* < 0.05) with minimum three proteins and maximum 500 proteins and provides the functional description for each complex. Using the MCODE algorithm, proteins and protein complexes that are enriched in the significantly dysregulated pathways were identified. The protein networks were visualized using Cytoscape (85) where nodes represent the proteins and edges represent the connections between the nodes. The size of the node in a complex shows the MCODE score, whereas the width of the edge shows the strength of the connection.

### Machine learning

The protein dataset with 512 tumour-enriched proteins was used as the input in this analysis. Because the number of patients is not large, a single train and test split of the dataset will lead to biased conclusions. Therefore, we decided to draw our conclusion based on results aggregated from multiple Monte Carlo runs of an XGBoost classifier with random train and test splits. We used 1,000 runs of Monte Carlo cross-validation on a random XGBoost classifier with 300 base learners and the rest of the hyperparameters set to defaults (Python package “XGBoost”). In each Monte Carlo run, the dataset was split randomly into 80% training and 20% test sets, stratified by the target variable GG (GG2 versus GG3). The test results from all the 1,000 runs were then aggregated, and the expected performance was reported.

## Data Availability

All protein measurements are available in Supplemental Data 2. The clinical and survival data can be downloaded from https://doi.org/10.7910/DVN/1RORIX by accepting the “Terms of Access for Restricted Files” agreement at Harvard Dataverse. The mapping file is in Supplemental Data 3. The raw mass spectrometry proteomic data and accompanying files have been deposited in the ProteomeXchange Consortium via the PRIDE (86) partner repository with the dataset identifier PXD041005.

Supplemental Data 2. Protein measurements of DIA-MS runs.

Supplemental Data 3. A mapping file for the clinical and survival data.

## Supplementary Material

Reviewer comments
