# Correction: Proteomic-based stratification of intermediate-risk prostate cancer patients

**DOI:** 10.26508/lsa.202503547

**Published:** 2025-12-01

**Authors:** Qing Zhong, Rui Sun, Adel T Aref, Zainab Noor, Asim Anees, Yi Zhu, Natasha Lucas, Rebecca C Poulos, Mengge Lyu, Tiansheng Zhu, Guo-Bo Chen, Yingrui Wang, Xuan Ding, Dorothea Rutishauser, Niels J Rupp, Jan H Rueschoff, Cédric Poyet, Thomas Hermanns, Christian Fankhauser, María Rodríguez Martínez, Wenguang Shao, Marija Buljan, Janis Frederick Neumann, Andreas Beyer, Peter G Hains, Roger R Reddel, Phillip J Robinson, Ruedi Aebersold, Tiannan Guo, Peter J Wild

**Affiliations:** 1 ProCan, Children’s Medical Research Institute , Faculty of Medicine and Health, The University of Sydney, Westmead, Australia; 2 iMarker Lab, Westlake Laboratory of Life Sciences and Biomedicine, Key Laboratory of Structural Biology of Zhejiang Province, School of Life Sciences, Westlake University , Hangzhou, China; 3 Institute of Basic Medical Sciences, Westlake Institute for Advanced Study, Hangzhou, China; 4 Urology and Nephrology Center, Department of Urology, Clinical Research Institute, Zhejiang Provincial People’s Hospital, People’s Hospital of Hangzhou Medical College, Hangzhou, China; 5 Department of Pathology and Molecular Pathology, University Hospital Zürich, Zürich, Switzerland; 6 Department of Urology, University Hospital Zürich, Zürich, Switzerland; 7 Department of Urology, Cantonal Hospital Lucerne, Lucerne, Switzerland; 8 IBM Zürich Research Laboratory, Zürich, Switzerland; 9 State Key Laboratory of Microbial Metabolism, Joint International Research Laboratory of Metabolic and Developmental Sciences, School of Life Sciences and Biotechnology, Shanghai Jiao Tong University, Shanghai, China; 10 Empa - Swiss Federal Laboratories for Materials Science and Technology, St. Gallen, Switzerland; 11 Swiss Institute of Bioinformatics, Lausanne, Switzerland; 12 CECAD, University of Cologne, Cologne, Germany; 13 Department of Biology, Institute of Molecular Systems Biology, ETH Zürich, Zürich, Switzerland; 14 Faculty of Science, University of Zürich, Zürich, Switzerland; 15 Goethe University Frankfurt, Dr. Senckenberg Institute of Pathology, University Hospital Frankfurt , Frankfurt am Main, Germany; 16 Frankfurt Institute for Advanced Studies, Frankfurt am Main, Germany

## Abstract

This study demonstrates the ability of a protein-based signature to risk-stratify prostate cancer patients with Gleason grade groups 2 and 3 and predict the risk of biochemical recurrence.

Article: Zhong Q, Sun R, Aref AT, Noor Z, Anees A, Zhu Y, Lucas N, Poulos RC, Lyu M, Zhu T, Chen G-B, Wang Y, Ding X, Rutishauser D, Rupp NJ, Rueschoff JH, Poyet C, Hermanns T, Fankhauser C, Rodríguez Martínez M, Shao W, Buljan M, Neumann JF, Beyer A, Hains PG, Reddel RR, Robinson PJ, Aebersold R, Guo T, Wild PJ (2023 Dec 4) Proteomic-based stratification of intermediate-risk prostate cancer patients. Life Sci Alliance 7(2): e202302146. doi: https://doi.org/10.26508/lsa.202302146. PMID: 38052461.

Following publication, the authors noticed an error in the Materials and Methods section. This section incorrectly referred to formalin fixed, paraffin-embedded (FFPE) tissue samples instead of fresh frozen (FF) samples. In the section “Biospecimen collection and pathology and clinical data,” the sentence “Tumour tissue samples were fixed with formalin and embedded with paraffin.” has been removed. In the section “Sample preparation and mass spectrometric acquisition,” the sentence “About 0.5 mg of FFPE tissue was punched from the sample, weighed, and processed for each biological replicate via the workflow as described previously (34).” has been changed to “About 0.5 mg of fresh-frozen prostate tissue was punched from the sample, weighed, and processed for each biological replicate via the workflow as described in Fig S7.”

Citation 34 remains because it is referenced elsewhere in the manuscript.

This error does not affect the conclusions of this manuscript.

