# Supplementary material for: Proteomic-based stratification of intermediate-risk prostate cancer patients
Source: Life Sci Alliance. 2023 Dec 4;7(2):e202302146. doi: 10.26508/lsa.202302146 (PMC10698198; doi:10.26508/lsa.202302146)
Supplement: Supplementary file 1 [file LSA-2023-02146_TableS1.docx]

Table S1. Clinicopathological features of patients with prostate cancer (PCa).

| No. of patients | 278 (277 tumour and 278 benign) | | |  |
| --- | --- | --- | --- | --- |
| No. of patients with follow-up | 198 | | |  |
| Median follow-up (months) | 59 | | | |
| Range (months) | 0-93 | | | |
| **Clinicopathological characteristics** | | | |  |
| **Variable** | | **n** | **%** |  |
| Age at diagnosis (median = 64 years, range = 41-83) | | | |  |
| <64 | | 132 | 47.6 |  |
| ≥ 64 | | 145 | 52.3 |  |
| Gleason grade | | | |  |
| GG1 | | 15 | 5.4 |  |
| GG2 | | 134 | 48.3 |  |
| GG3 | | 70 | 25.2 |  |
| GG4 | | 29 | 10.4 |  |
| GG5 | | 29 | 10.4 |  |
| Tumour stage (pT) | | | |  |
| pT1 | | 159 | 57.4 |  |
| pT2 | | 79 | 28.5 |  |
| Unknown | | 39 | 14 |  |
| Surgical margin | | | |  |
| Negative | | 145 | 52.3 |  |
| Positive | | 93 | 33.5 |  |
| Unknown | | 39 | 14 |  |
| Tumour percentage | | | |  |
| 40% | | 1 | 0.36 |  |
| 50% | | 13 | 4.7 |  |
| 60% | | 28 | 21.6 |  |
| 70% | | 78 | 25.2 |  |
| 80% | | 136 | 28.8 |  |
| 90% | | 21 | 32.4 |  |
| Biochemical recurrence events | | | |  |
| Missing | | 78 | 28.1 |  |
| No | | 168 | 60.6 |  |
| Yes | | 31 | 11.1 |  |
