# Supplementary material for: Proteomic-based stratification of intermediate-risk prostate cancer patients
Source: Life Sci Alliance. 2023 Dec 4;7(2):e202302146. doi: 10.26508/lsa.202302146 (PMC10698198; doi:10.26508/lsa.202302146)
Supplement: Supplementary file 2 [file LSA-2023-02146_TableS2.docx]

Table S2. Summary of 39 Signature Proteins.

| Proteins | Biomarkers |
| --- | --- |
| FTSJ3 | Breast cancer progression (PMID: 31957540) |
| CALD1 | Prognostic biomarker of gastric cancer (PMID: 34189308), is an Androgen related gene (ref 50) and potential diagnostic biomarker for PCa (ref 46) |
| RRP9 | Gemcitabine resistance in pancreatic cancer (PMID: 36434608) |
| AGR3 | Prognostic biomarker of breast cancer (PMID: 25875093) |
| MRPS22 | Associated with Prostate cancer poor prognosis (ref 64) |
| F5 | Prognostic biomarker of gastric cancer (PMID: 32190689), associated with increased risk of breast cancer (ref 66), FDA approved drug target |
| ACSS3 | Bladder cancer progression (PMID: 32398651),High-grade ovarian cancer (PMID: 35502666) |
| PDF | Prognostic in head and neck, cervical, renal and lung cancer (Human protein atlas data ref 39) |
| MRPL40 | Prognostic in Cervical cancer (HPA ref 39) |
| PPAT | Prognostic biomarker for hepatocellular carcinoma (PMID: 35027843) |
| GCNT1 | Prostate cancer (PMID: 15932919, 26768364, 24854630) |
| MUC2 | Potential prognostic in Colon cancer (PMID: 31121192) and pancreatic cancer (PMID: 31590960) |
| RPF2 | Prognostic in renal, head and neck and liver cancer (HPA ref 39) |
| PSMG3 | Prognostic in liver cancer (HPA ref 39) |
| PPP1R14B | A prognostic and immunological biomarker in pan-cancer (PMID: 34858476) |
| ASPN | Prostate cancer progression (PMID: 28152543) |
| SMYD3 | Prostate cancer, breast cancer, colon cancer, colorectal cancer, ovarian cancer, and hepatocellular carcinoma (PMID: 30544196, 29969917, 31417652, 26908355, 25980436, 34301921) |
| ECH1 | Lung cancer (PMID:28638733) |
| ANXA4 | FDA approved drug target (ref 39), androgen regulated gene (ref 50) |
| TMEM126B | Potential drug target (ref 39) |
| EARS2 | Potential drug target (ref 39) |
| MYLK | Potential drug target (ref 39), androgen regulated gene (ref 50) |
| PUM3 | Androgen regulated gene (ref 50) |
| TPM1 | Androgen regulated gene (ref 50) |
| HNRNPA2B1 | Associated with Prostate cancer poor prognosis (ref 64) |
| MYL9 | Prognostic biomarker of prostate cancer (PMID: **24338276)** |
| CSRP1 | Survival prediction and therapeutic response in prostate cancer (PMID: 35143037) |
| SMTN | Neoadjuvant chemotherapy prediction biomarker (PMID: **35928032**) |
| MTHFD1L | Potential treatment targeted for hepatocellular carcinoma (PMID: 28394261) |
| SYNM | No report |
| ADH5 | No report |
| FBLN5 | Tumor suppressor in the lung cancer (PMID: **17929269**) |
| SYNPO2 | Tumor suppressor in the many cancers, including prostate cancer (PMID: 37544991) |
| EFEMP1 | Potential biomarker for prostate cancer diagnosis (PMID: **21571867**) |
| DES | Biochemical-free recurrence prediction biomarker for prostate cancer (PMID: **14581350**) |
| PDLIM7 | No report |
| HSPB6 | No report |
| TAGLN | Tumor suppressor in the prostate cancer (PMID: **33771884**) |
| LGALS1 | Potential target of prostate cancer (PMID: **23108139**) |
| TOR2A | No report |
| MRPS14 | No report |
